# Clinical and Histopathological Predictors of Survival in Regional Lymph Node Metastases From Cutaneous Squamous Cell Carcinoma of the Head and Neck: A Retrospective Analysis

**DOI:** 10.7759/cureus.100599

**Published:** 2026-01-02

**Authors:** Esra Izeldin

**Affiliations:** 1 Oral and Maxillofacial Surgery, East Kent Hospitals University NHS Foundation Trust, Ashford, GBR

**Keywords:** cutaneous squamous cell carcinoma, recurrence, regional adenopathy, skin cancer, survival outcomes

## Abstract

Background: Cutaneous squamous cell carcinoma (cSCC) is a common skin cancer, with a subset demonstrating aggressive behaviour leading to regional lymph node metastases and poor prognosis. This study evaluated the clinical characteristics, prognostic factors, and survival outcomes in patients with nodal metastases from head and neck cSCC.

Methods: A retrospective analysis was performed on 19 patients with histologically confirmed nodal metastases from cSCC treated between 2018 and 2023 at East Kent Hospitals NHS Trust. Data on patient demographics, tumour features, treatment, and follow-up were reviewed. Survival outcomes were estimated using Kaplan-Meier analysis, and univariate Cox regression identified potential prognostic factors for disease-free survival (DFS) and overall survival (OS).

Results: The mean patient age was 84.3 years, with a male predominance (84.2%). Primary tumours were most frequently located on the scalp and cheek and were predominantly moderately or poorly differentiated. High-risk features included tumour size >2 cm, perineural invasion (36.8%), and extracapsular extension (52.6%). All patients underwent neck dissection, with 47% requiring additional parotidectomy; 94.7% received adjuvant radiotherapy. Despite this, the majority developed recurrence within 11 months. The five-year OS was 21.1%, and DFS was 5.3%. Tumour size was significantly associated with reduced DFS (p=0.026), while other pathological variables showed no independent effect.

Conclusions: Metastatic cSCC of the head and neck carries a poor prognosis, even with aggressive multimodal therapy. High-risk pathological features remain key predictors of recurrence. Improved risk stratification and incorporation of novel systemic therapies may help improve long-term outcomes in this patient group.

## Introduction

Cutaneous squamous cell carcinoma (cSCC) is the most frequently diagnosed malignancy among individuals of Caucasian descent and represents the most common cancer in the white population of the United Kingdom and globally [1]. Its incidence is rising both in the United Kingdom and internationally, currently estimated at 31.4 cases per 100,000 people annually. This increase is largely associated with greater exposure to ultraviolet radiation (UVR) and an ageing population. Additionally, the states of immunosuppression, whether due to underlying conditions such as organ transplantation or malignancy or as a result of immunosuppressive treatments including chemotherapy, biologic agents, and conventional immunosuppressive drugs, contribute to the elevated risk of developing cSCC [2]. 

Unlike basal cell carcinoma (BCC), cSCC has the potential to metastasise to both regional lymph nodes and distant organs. The reported incidence of metastasis ranges from 2% to 5%, and when present, it is often associated with a significantly poorer prognosis, with survival typically limited to 2-5 years. Lymph node involvement in cSCC increases the likelihood of both recurrence and mortality by approximately 30%. Despite this, the overall prognosis for cSCC remains favourable, with an estimated five-year cure rate of around 90%. Notably, 75% of recurrences occur within the first two years of diagnosis, and 90% are observed within five years [2].

Several factors contribute to the high-risk profile of cSCC, including tumour size greater than 2 cm, depth of invasion exceeding 4 mm, poor histological differentiation, presence of perineural invasion (PNI) or lymphovascular invasion (LVI), immunosuppressed status, and primary tumour location on high-risk sites such as the ear or lip. While metastatic spread often remains confined to regional lymph nodes, the prognosis is guarded, with reported cure rates as low as 34.4% in these high-risk cases [3].

The first line of treatment for cSCC is wide local excision to enable the confirmation of diagnosis and assessment of the surgical margin. A surgery margin of 4 mm is sufficient if the tumour size is less than 2 cm; for tumours more than 2 cm, 6-10 mm is considered safe. The location of cSCC can determine whether the patient is at high risk (e.g., ear and lip).

Adjuvant radiotherapy should be considered if there's PNI and the surgical margin of the tumour is not free of disease and for all patients with positive lymph node involvement and extracapsular involvement. Chemotherapy is considered in stage IV cSCC. Patients with metastatic cSCC have a poor prognosis, with an overall survival (OS) rate of 34.5% [2].

The purpose of this study is to characterise the clinical and histopathological presentation and prognostic factors in patients with regional and lymph node metastasis and the disease survival-free rate.

## Materials and methods

We conducted a retrospective analysis of 19 patients with histologically confirmed regional lymph node metastasis from cSCC of the head and neck. All patients underwent neck dissection, with or without parotidectomy, between 2018 and 2023 at East Kent Hospitals NHS Trust, England.

Inclusion criteria

Included were patients diagnosed with cSCC of the head and neck with regional lymph node metastasis (parotid and/or cervical nodes) between 2018 and 2023 who underwent curative-intent neck dissection±parotidectomy.

Exclusion criteria

Excluded were patients with mucosal squamous cell carcinoma, BCC, or Merkel cell carcinoma, patients treated with palliative intent, and patients with less than six months of follow-up.

Data collection

Clinical and pathological data were extracted from multidisciplinary team (MDT) meeting notes, imaging reports, histopathology records, and electronic medical records (Sunrise system).

Collected variables included patient characteristics (age, sex, comorbidities (including immune status), and prior treatment), tumour characteristics (primary site, histological differentiation, margin status, LVI, PNI, extranodal extension (ENE), number of positive lymph nodes, and size of the largest nodal metastasis), adjuvant treatment (radiotherapy (including dose and timing), chemotherapy, and immunotherapy), and follow-up (time from surgery to the last follow-up or death and details on recurrence confirmed by biopsy, radiologic imaging, or clinical examination). All patients underwent wide local excision of the primary tumour, aiming for negative margins. High-risk features included immunosuppression, tumour size >2 cm, PNI, and recurrent disease.

Statistical analysis

Disease-free survival (DFS) and OS were the primary outcomes. Survival durations were measured from the date of surgery.

Univariate and multivariate analyses were performed using Cox proportional hazards regression to evaluate potential predictors of survival, including primary tumour site, site of nodal metastasis, number of involved lymph nodes, extent of nodal involvement, size of nodal metastasis (>3 cm vs. <3 cm), and presence of ENE.

Kaplan-Meier survival estimates were used to evaluate the effect of clinical and pathological variables on DFS. Statistical significance was defined as p<0.05. All patients were staged according to the 7th edition of the American Joint Committee on Cancer (AJCC) staging system. The endpoint for analysis was DFS, calculated from the date of surgery to the date of first disease recurrence or death from any cause.

## Results

The primary cSCC was divided into five different anatomical sites (scalp, lip, cheek, forehead, and ear). The size of cSCC is categorised into <2 cm, between 2 and 4 cm, and >4 cm. The tumour differentiation was categorised into well, moderately, and poorly differentiated. The depth of the lesion was categorised histologically between <1 mm, 1-2 mm, 2-4 mm, and >4 mm.

During the period of follow-up, primary lesion patients with regional metastasis were identified, and the site and time of metastasis were documented. The site of metastasis was documented based on anatomic region (parotid, neck levels I-V).

A cohort of 19 patients was included in the study, comprising three females (15.8%) and 16 males (84.2%), aged between 68 and 95 years, with a mean age of 84.3 years. All patients had a mean follow-up of 40.8 months, ranging between 12 and 60 months. Sixteen (84.2%) patients of our cohort were immunocompetent, and a minority of three patients (15.8%) were immunocompromised (azathioprine, type 2 diabetes mellitus, and a previous history of prostate cancer) (Table 1).

**Table 1:** Patient demographics Values are presented as n (%) or mean (range). p<0.05 was considered statistically significant.

Number of patients	Age (mean, range)	Sex (male/female)	Immunocompetent	Immunocompromised
19	84.3	16 (84.2%)/3 (15.8%)	16 (84.2%)	3 (15.8%)

Primary lesion characteristics

All 19 patients underwent wide local excision of the primary tumour. The most documented primary sites were the scalp (seven, 36.8%) and cheek (four, 21.1%), followed by the lip (three, 15.8%) and auricular region (one, 5.3%) and one case (5.3%) with an unknown primary (Table 2). The median tumour size was 2.35 cm (range: 1-4 cm), and the mean histological depth was 3.6 mm (range: 0.3-9.5 mm). Histologically, 17 (89.5%) tumours were moderately to poorly differentiated squamous cell carcinoma, one (5.25%) was well differentiated, and one case (5.25%) lacked documented differentiation. Perineural invasion was identified in seven (36.8%) tumours, and lymphovascular invasion was present in four (21.1%) (Table 2).

**Table 2:** Primary tumour characteristics Values are presented as n (%) or median (range) and mean±SD where appropriate. p<0.05 was considered statistically significant.

Characteristic	Value
Site	
Scalp	7 (36.8%)
Cheek	4 (21.1%)
Lip	3 (15.8%)
Auricular region	1 (5.3%)
Unknown	1 (5.3%)
Median tumour size (cm)	2.35
Mean depth (mm)	3.6
Differentiation	
Moderately to poorly differentiated	17 (89.5%)
Well differentiated/not documented	2 (10.5%)
High-risk features	
Perineural invasion	7 (36.8%)
Lymphovascular invasion	4 (21.1%)

During follow-up, 12 (63.2%) patients developed regional lymph node or neck metastases, with a mean time to recurrence of 11 months (range: 12-36 months). Of these, 26.3% developed metastases within 12 months and 10.5% after 24 months. 

Location of the nodes

The majority of metastatic lymph nodes (six, 31.6%) were located in level II, followed by level I in five (26.3%) and level V in three (15.8%). Additionally, five (26.3%) patients presented with involvement of multiple nodal levels. The mean size of the largest metastatic lymph node was 2.56 cm (range: 0.56-5 cm). Extracapsular extension (ECE) was identified in 52.6% of patients (n=10), whereas nine (47.4%) patients had no documented or absent ECE (Table 3).

**Table 3:** Nodal metastases and treatment data Values are presented as n (%) or mean±SD. Statistical comparisons were performed using the chi-squared test. p<0.05 was considered statistically significant.

Characteristic	Value
Level I	5 (26.3%)
Level II	6 (31.6%)
Level V	3 (15.8%)
Multiple levels	5 (26.3%)
Mean number of positive nodes	4.26
Extracapsular extension	10 (52.6%)
Adjuvant radiotherapy	18 (94.7%)

Surgery

All 19 patients with nodal metastases underwent neck dissection. Of these, 10 patients (52.6%) underwent combined neck dissection with parotidectomy, including two patients (10.5%) who had total parotidectomy and eight patients (42.1%) who had superficial parotidectomy. Eleven patients (57.9%) underwent neck dissection alone. Selective neck dissections were performed in 15 patients (78.9%), involving levels I-IV, II-V, or I-V. Four patients (21.1%) required modified radical neck dissection due to disease extent. Flap reconstruction was performed in 15 patients (78.9%), representing the majority of the cohort requiring soft tissue reconstruction following neck dissection.

Number of nodal metastases

The mean number of metastatic lymph nodes identified was 4.26 (range: 0-20) (Table 3), with the highest number of nodal metastases recorded being 20. The mean number of lymph nodes removed was 46.7 (range: 14-75), with a mean of 4.26 positive nodes per patient (range: 0-20). The mean size of the largest nodal metastasis was 41.1 mm (range: 11-80 mm). Regarding surgical margins, more than half of the patients achieved clear margins, approximately 30% had close margins (≤0.5 mm), and 10.5% had positive margins.

Radiation

Adjuvant radiotherapy was administered to 19 patients based on the presence of high-risk features, including tumour size >2 cm, multiple nodal involvement, perineural invasion, immunosuppression, and close or positive surgical margins. Of these, 18 patients (94.7%) received external beam radiotherapy, while one patient declined treatment. The delivered radiation dose ranged from 55 to 66 Gy over a four-week period. The primary indication for adjuvant radiotherapy was nodal positivity. Additionally, two patients received concurrent chemoradiotherapy, and one patient underwent immunotherapy as part of the adjuvant treatment regimen.

Locoregional and distant recurrence

Most patients developed regional lymph node metastases within the first year following primary tumour surgery. The mean time from treatment of the primary tumour to the development of clinically evident regional nodal disease was 11 months (range: 12-36 months). Two patients experienced local recurrence at the primary tumour site. Three patients developed recurrence despite surgery and adjuvant radiotherapy: one presented with locoregional recurrence on the cheek, another developed regional metastasis to the floor of the mouth (primary site: lower lip), and a third developed regional metastasis to the clavicular region post-surgery. Two patients experienced distant metastases to the lungs. One of these cases was associated with recurrence at the primary scalp site and subsequent pulmonary spread. Another patient had local recurrence in the auricular region, accompanied by distant lung metastases.

Survival

At a median follow-up of 34.2 months (range: 6-72 months), the five-year OS rate was 21.1%, while the five-year DFS rate was 5.3%. The mean DFS duration was 21.6 months (range: 8-60 months), indicating a high rate of recurrence or disease progression within the first few years after treatment (Table 4).

**Table 4:** Survival outcomes Values are presented as percentages or mean (range). Survival analysis was performed using the Kaplan-Meier method. p<0.05 was considered statistically significant.

Outcome	Value
5-year overall survival	21.1%
5-year disease-free survival	5.3%
Mean disease-free survival	21.6 months (8-60)
Median time to recurrence	11 months (12-36)

Analysis

Univariate survival analysis demonstrated that tumour size was significantly associated with DFS. Patients with tumours larger than 2 cm experienced significantly shorter DFS compared to those with smaller tumours (p=0.049). However, tumour size did not show a significant association with OS (p=0.410).

ECE did not significantly affect DFS (p=0.375) or OS (p=0.416). Similarly, receipt of adjuvant radiotherapy was not associated with DFS (p=1.000) or OS (p=0.416).

Immunosuppression was not significantly associated with DFS (p=0.225), although a borderline association was observed with OS, with immunosuppressed patients tending towards worse outcomes (p=0.079).

Neither perineural invasion nor tumour differentiation showed a statistically significant association with DFS (p=0.610 and p=0.574, respectively) or OS (p=0.198 and p=0.171, respectively) (Table 5). 

**Table 5:** Univariate survival analysis (log-rank test results for DFS and OS) Values are log-rank χ² statistics with corresponding p-values comparing Kaplan-Meier survival curves between groups. "NS" indicates not significant or not testable due to the small sample size. Multivariate Cox regression could not be reliably performed due to cohort size and predictor separation; therefore, only univariate analyses are presented. p<0.05 was considered statistically significant. DFS: disease-free survival; OS: overall survival

Predictor	DFS log-rank χ²	DFS p-value	OS log-rank χ²	OS p-value
Tumour size >2 cm	3.861	0.049	0.678	0.410
Tumour location	-	0.059	-	NS
Differentiation (moderate/poor vs. well)	0.315	0.574	1.870	0.171
Perineural invasion	0.260	0.610	1.657	0.198
Lymphovascular invasion	-	NS	-	NS
Tumour depth	-	NS	-	NS
Extracapsular extension	0.788	0.375	0.663	0.416
Radiotherapy received	0.000	1.000	0.663	0.416
Immunosuppressed	1.473	0.225	3.078	0.079

## Discussion

In this series of 19 patients with metastatic cSCC of the head and neck, we observed a high incidence of regional lymph node metastases and early recurrence despite surgery and adjuvant therapy. Most patients developed nodal disease within the first postoperative year, underscoring the aggressive nature of high-risk cSCC.

Univariate survival analysis revealed that tumour size was the only factor significantly associated with DFS. Patients with tumours >2 cm experienced shorter DFS compared to those with smaller tumours (log-rank χ²=3.861; p=0.049). Tumour size, however, was not associated with OS (χ²=0.678; p=0.410). Tumour location demonstrated a borderline association with DFS (p=0.059) but no impact on OS. Other pathological features, including differentiation (DFS: χ²=0.315 and p=0.574; OS: χ²=1.870 and p=0.171), PNI (DFS: χ²=0.260 and p=0.610; OS: χ²=1.657 and p=0.198), LVI, tumour depth, and ECE, were not significantly associated with DFS or OS in this cohort. Similarly, adjuvant radiotherapy and immunosuppression did not show statistically significant effects, although immunosuppressed patients tended toward worse OS (χ²=3.078; p=0.079).

These findings contrast with much of the existing literature, which has identified a broader set of high-risk features. Harris et al. reported that lesions located on the scalp, ear, or cheek and those with PNI or poor differentiation were significantly more likely to recur or metastasise [4]. Feinstein et al. reported comparable findings in their retrospective analysis [5]. Szewczyk et al., in a series of 100 patients, found that tumour differentiation, size, and anatomical location were predictive of neck metastases [6]. Trosman et al. identified PNI, tumour size >2 cm, and positive margins as significant predictors of nodal disease [7]. Thompson et al., in a systematic review, concluded that tumour depth >6 mm, poor differentiation, and PNI were strongly associated with metastases [8]. The absence of statistical significance for these variables in our series likely reflects our small sample size, which reduced power to detect these associations, rather than a true absence of effect.

Nodal burden also appeared clinically relevant in our series, although formal statistical analysis was limited. The mean number of positive lymph nodes was 4.26, and patients with higher nodal burden tended to have shorter DFS. These observations are consistent with Sood et al. [9], who found that increasing nodal involvement correlated with distant metastases and poorer DFS. Multiple studies have similarly confirmed that nodal burden is a strong prognostic factor for both DFS and OS.

The role of adjuvant radiotherapy remains debated. In our study, nearly all patients received postoperative radiotherapy, and we did not observe a survival benefit (DFS: χ²=0.000 and p=1.000; OS: χ²=0.663 and p=0.416). By contrast, Harris et al. [4], Warren et al. [10], and Mifsud et al. [11] reported that adjuvant radiotherapy or chemoradiation reduced recurrence and improved OS in high-risk cSCC. Conversely, Khurana et al. found high recurrence rates despite surgery and radiotherapy, particularly in patients with positive margins [12]. The discrepancy between our findings and prior reports likely reflects our cohort size and the fact that most patients with adverse features received radiotherapy, limiting our ability to detect differences.

Immunosuppression is a recognised adverse prognostic factor in cSCC. In our study, immunosuppression was not significantly associated with DFS (χ²=1.473; p=0.225) but trended toward worse OS (χ²=3.078; p=0.079). This aligns with Tam et al. [13], who concluded that immunosuppressed patients have inferior outcomes. In contrast, Sun et al. [14] reported that recurrence after surgery and radiotherapy predicted poor prognosis regardless of immune status. Our small sample size may explain why the effect of immunosuppression did not reach significance.

In our cohort, ECE was present in 52.6% of patients but was not significantly associated with DFS or OS (DFS: χ²=0.788 and p=0.375; OS: χ²=0.663 and p=0.416). Larger studies have consistently reported ECE as one of the strongest predictors of poor outcomes. For instance, Luchini et al., Leeman et al., and Newman et al. [15-17] found that nodal ECE was associated with reduced five-year survival and elevated recurrence risk, even after surgery and radiotherapy. The lack of statistical association in our series again reflects its limited size.

OS in our cohort was poor, with five-year OS at 21.1% and DFS at 5.3%, consistent with prior reports [18,19]. Wackel et al. reported five-year OS below 15% in advanced nodal/distant cSCC [18], while Beach et al. showed nodal involvement reduces five-year OS by ~50% compared with localised disease [19]. By contrast, early-stage cSCC treated with surgery alone achieves a five-year survival exceeding 99% [20]. These comparisons underscore the devastating prognostic impact of nodal metastases and high-risk pathological features.

Limitations

This study is limited by its retrospective design, its small sample size, and the heterogeneity of treatment. These factors reduce statistical power and limit generalisability. Nevertheless, our data reinforce tumour size as the only reproducible prognostic factor in this cohort and highlight the importance of larger, multi-institutional studies to clarify the roles of ECE, PNI, LVI, immunosuppression, and adjuvant radiotherapy in metastatic cSCC.

## Conclusions

In patients with metastatic cSCC of the head and neck, tumour size was the only factor significantly associated with DFS, while other histopathological features, such as ECE, PNI, and differentiation, did not independently influence outcomes in this small cohort. OS was not significantly affected by any single variable, although immunosuppression showed a borderline adverse effect. These findings underscore the prognostic value of tumour size in guiding risk stratification while highlighting the need for larger, multi-institutional studies to better define the impact of additional pathological features on survival.

Early detection, meticulous risk stratification, and prompt surgical and adjuvant management remain critical to improving outcomes. Future studies should focus on integrating novel systemic therapies, including immunotherapy, to address the high recurrence and mortality rates in advanced cSCC.
